# External clinical audits in clinical radiology in Finland

**DOI:** 10.1093/rpd/ncaf182

**Published:** 2026-01-09

**Authors:** Petro Julkunen, Nina Heinola, Taina Autti, Ritva Bly

**Affiliations:** Department of Technical Physics, University of Eastern Finland, Yliopistonranta 8, FI-70210 Kuopio, Finland; Diagnostic Imaging Center, Kuopio University Hospital, Puijonlaaksontie 2, FI-70210 Kuopio, Finland; Labquality Oy, Kumpulantie 15, FI-00520 Helsinki, Finland; HUS Medical Imaging Center, Radiology, University of Helsinki and Helsinki University Hospital, P.O. Box 340, FI-00290 Helsinki, Finland; Radiation and Nuclear Safety Authority (STUK), P.O. Box 14, FI-00811 Helsinki, Finland

## Abstract

Clinical audits have been implemented in Finland to healthcare organizations over 20 years. We introduce the outcomes of fourth round external clinical audits on medical radiological procedures following the implementation of the EU Directive (Basic Safety Standards). We analyzed 31 external clinical audits on radiological practices in 2018–2022. The summarized strengths and recommendations from the audits were collected from the main auditing organization’s database. 306 recommendations were given, 18% related to definitions of responsibilities, 16% to internal procedural guidelines, 16% to personnel education, and 25% to quality assurance and utilization of self-assessments of radiological procedures. Private and small organizations received less recommendations than large and public operators. Moreover, 215 strengths were reported, focusing on the clarity of responsibilities (21%) and excellence in quality assurance (15%). Reaching and maintaining feasible radiological diagnostic accuracy by consideration of radiation safety requires continuous quality assurance and development facilitated, and monitored by clinical audits.

## Introduction

United Nations Scientific Committee on the Effects of Atomic Radiation estimated that in 2022 there were about 7.3 billion radiological examinations or procedures conducted globally annually [1], while in Finland, the corresponding number is about 7.7 million [2]. This number of radiological examinations involves a collective radiation dose that in the scale of populations requires continuous optimization and justification to minimize any adverse effects from radiation while considering the individual patient needs for such examinations to benefit their health.

Clinical audit in radiology is a systematic evaluation of radiological practices and procedures set to improve the quality of patient care [3–6]. It is a multidisciplinary effort conducted by team of experts, and an integral part of the quality management system [4, 5]. It plays a crucial role in ensuring that radiation exposure is kept as low as reasonably achievable (ALARA-principle) for patients, staff, and general population [5, 7]. As an outcome, the clinical audits improve the quality of patient care, enhance the effective use of resources, and provision and the supply of clinical services, and advance professional education and training [4].

Clinical audits have been stipulated in Finnish legislation since year 2000 and have been implemented ever since to both public and private healthcare organizations. Clinical audits are stipulated in Finland to include annual self-assessments, internal audits, and external audits [8]. Here we focus on external clinical audits of radiological diagnostics and represent the routine and outcomes from the audits conducted in Finland. The authority enforcing and supervising the abidance of the legislation in this aspect is the Radiation and Nuclear Safety Authority (STUK), while the operators of radiation in clinical radiological use are obligated to enforce all forms of the clinical audit. According to the Finnish Radiation Act [8], external clinical audit refers to a planned evaluation of the medical use of radiation, in which examination and treatment practices are followed, radiation exposures, and examination and treatment results are explained. The practices are then compared with proven practices [5, 6] and measures assessed to develop practices and prevent unjustified radiation exposure. External clinical audits are carried out according to the graded approach depending on the risk level of the practice, in 6–8 year periods by an experienced and competent multidisciplinary team of external experts [4], in diagnostics typically including a radiographer, a radiologist and a medical physicist. Low-level radiation risk practices, such as intraoral dental radiological practices, are exempted from external clinical audits. The external clinical audits are currently on the fourth cycle. The clinical audits are conducted by companies specializing in audits under different standards while remaining independent from the audited organizations and regulatory bodies. Regulatory inspections are conducted by STUK to verify compliance with the regulatory requirements [5, 9], while the clinical audits focus on structure and processes [4]. The auditing companies nominate the auditing team that carries out the external clinical audit. The audit team will be approved by the audited organization prior to the audit.

The external clinical audits in Finland follow the guidelines of the Finnish advisory committee for clinical audit (KLIARY) appointed by the Finnish institute for health and welfare. KLIARY is an expert group independent of auditing organizations formed to coordinate and develop auditing activities and to evaluate the audit programs. It provides recommendations to guide the selection of external clinical audit priorities and procedures. The organizing of the Finnish external clinical audits contain: (i) identification of the requirement and external clinical audit periods based on the risk level of radiation practices, (ii) selection of auditing organization, (iii) scheduling audit, (iv) evaluating audit team expertise requirements, (v) nominating the auditing team, (vi) pre-approving the team, (vii) collecting data on prior audits (internal and external), self-assessments, normal operational characteristics, responsibilities, procedural instructions, etc., (viii) conducting audit, and (ix) reporting findings.

The external clinical audit focuses on reviewing the practices, and provides feedback as strengths and recommendations. The report goes to the audited organization, not to authorities, but is available for regulatory control on radiation safety [5]. The thematic focus during the current auditing period 2018–2022 (the fourth cycle of external clinical audits) has been in CT procedures (paediatric CT of head, paediatric trauma CT, and adult CT of abdomen) and in small radiological units on lumbar spine imaging, dental panoramic tomography, and paediatric imaging.

An EU-wide QuADRANT project, funded by the European Commission’s (EC) Directorate General for Energy, aims to support in implementing the Basic Safety Standards Directive (BSSD) and to advance quality and safety of medical radiation applications. It focuses on the implementation of clinical audits as mandated in the BSSD to identify good practices, and develop guidance and recommendations. The project has resulted in a new radiation protection recommendation [9] under the EC on “*Current Status and Recommendations for Improving Uptake and Implementation of Clinical Audit of Medical Radiological Procedures*”, and should eventually result in leaner and more unified clinical audit practices in Europe. Heads of the European Radiological Protection Competent Authorities have made a position paper on the Clinical Audits in Medical Radiological Practices [10] to tackle some identified gaps in general understanding on how the set regulatory requirements should be met.

Paralleling with the advancement of the clinical audit procedures in Europe, here we introduce the outcomes of 31 fourth cycle external clinical audits on medical radiological procedures in Finland following the national implementation of the EU Council Directive of BSSD [3], since 2018 [8]. With this report, we hope to provide a structured analysis of the type of observations and remarks that are generally made during the external clinical audits and characterize and compare those between different operators.

## Materials and methods

### Data

We analyzed retrospectively summaries from all external clinical audits on radiological practices in 2018–2022 conducted to 31 radiological operators consisting of altogether 56 audited radiological units. The summaries were taken from Labquality Oy’s database. Labquality Oy (Helsinki, Finland) conducts external clinical audits, e.g. in radiological procedures. The information gathered from the database included grouping into public and private sector operators, as well as, in parallel to small and large radiological operators. Of the 19 public sector operators, 14 were considered as large radiological operators and 5 small radiological operators, and of the 12 private sector operators, all were considered as small radiological operators. A *Small* radiological operator is defined here as an operator that mainly performs conventional diagnostic radiology at a low volume of less than 5000 examinations per year, or a unit with about 3 radiographers with less than 3500 examinations per year per radiographer. The other operators were *large* operators including one or more audited units.

From the 31 external clinical audits conducted on radiological operators, the information that was gathered anonymously was the type of feedback (recommendation or identified strength), its categorized description (Table 1), number of audited units 1–5 within the operator, the sector of operation (public/private), and operator size (small/large). Small operators included 1–3 audited units/operator with overall 27 audited units, while the large operators included 1–5 audited units/operator with overall 29 audited units.

**Table 1:** Assessed criteria during external clinical audits.

**Criterion index**	**Description**
1	Realization of defined authorizations and responsibilities in practice
2	The practice and flow of information followed in the justification assessment
3a	Instructions and practices for execution of examinations, treatment and procedures causing exposure, and ensuring that the treatment is carried out according to the plan
3b	Optimal and appropriate use of examination and treatment equipment
3c	Optimization of dose and image quality resulting from medical exposure
3d	The quality of the statement about the examination
4	The achieved examination and treatment outcomes and the flow of information
5	Personnel training
6	Quality assurance, results of operational self-assessments and use of results
11	Consideration of the recommendations from the previous audits

### Statistical analysis

The number of recommendations and strengths were compared between the public and private, and between the small and large radiological operators within different evaluation criteria (Table 1) using Wilcoxon rank sum test. Correlation matrix was built to screen for connections between different criteria in the number of identified recommendations and strengths. Correlation analyses were conducted using Pearson’s linear correlation coefficient (*r*).

## Results

**Figure 1:**
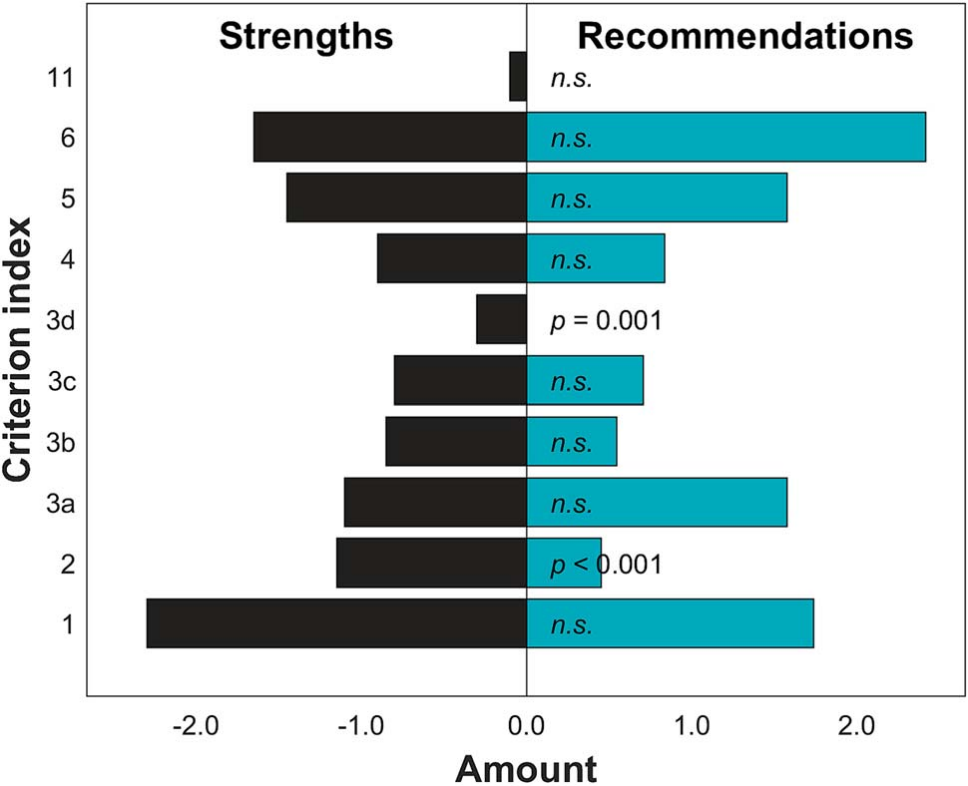
The average number of strengths and recommendations of all operators within each evaluated criterion. The bars indicate the average number of strengths identified or recommendations received per operator. Significant differences have been indicated with the *P*-values shown; while the non-significant differences have been indicated with *n.s.* the criterion index in the vertical axis refers to those shown in Table 1.

The summarized findings on recommendations and identified strengths given in the audits are shown in Table 2. Comparing the number of recommendations to strengths revealed that overall, there were no differences in their numbers (*P* = .734). Within the categorized descriptions, more strengths were identified than recommendations given in categorized criteria 2 (*the practice and flow of information followed in the justification assessment*) and 3d (*the quality of the statement about the examination*) (*P* ≤ .001; Fig. 1). Overall, large operators received significantly more recommendations than the small operators (*P* = .011), and the public operators received more recommendations than the private sector operators (*P* = .011). No differences were observed in the overall number of strengths between the sectors (*P* = .123) or different size operators (*P* = .574).

**Table 2:** Summary statistics.

**Parameter**		**All operators**	**Public sector**	**Private sector**	**Large operators**	**Small operators**
Number of audited operators		31	19	12	14	17
Number of recommendations^*^	total average range	306 9.9 2–19	224 11.8 8–19	82 6.8 2–12	175 12.5 8–19	131 7.7 2–14
Number of strengths	total average range	212 10.6 2–21	90 10.0 3–21	122 11.1 8–16	73 10.4 3–21	139 10.7 8–16

^*^Indicates significant difference (*P* < .05) between public and private operators, and between large and small radiological operators.

**Figure 2:**
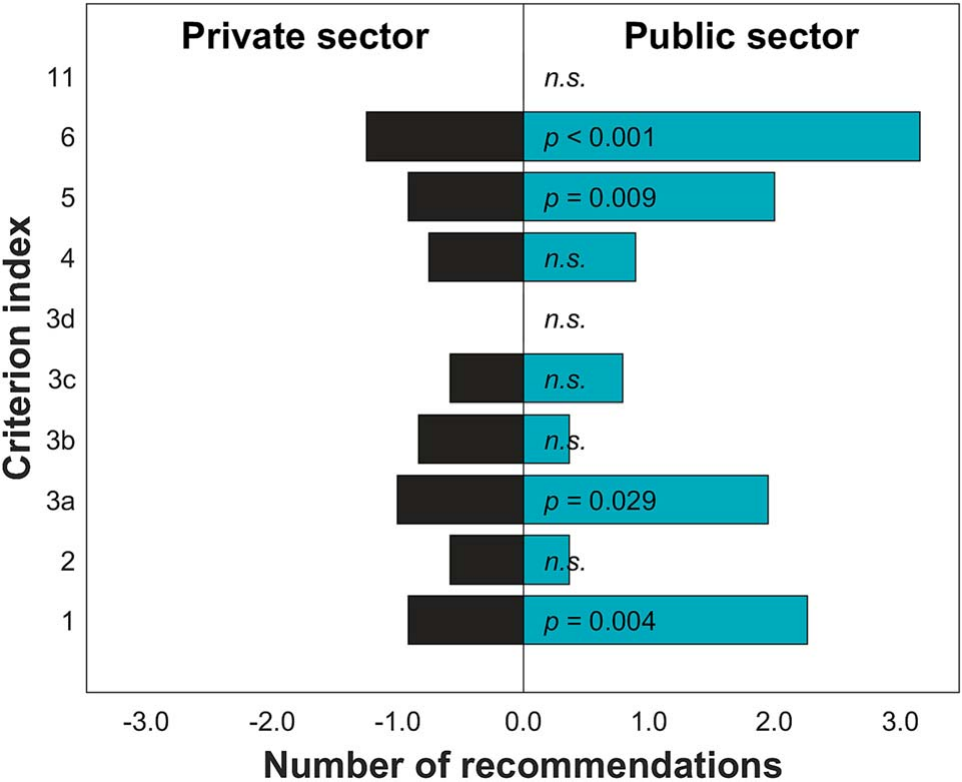
Comparison of the number of recommendations between the public and the private sector operators within each evaluated criterion. The bars indicate the average number of recommendations received per operator within the sector. Significant differences have been indicated with the *P*-values shown; while the non-significant differences have been indicated with *n.s.* the criterion index in the vertical axis refers to those shown in Table 1.

**Figure 3:**
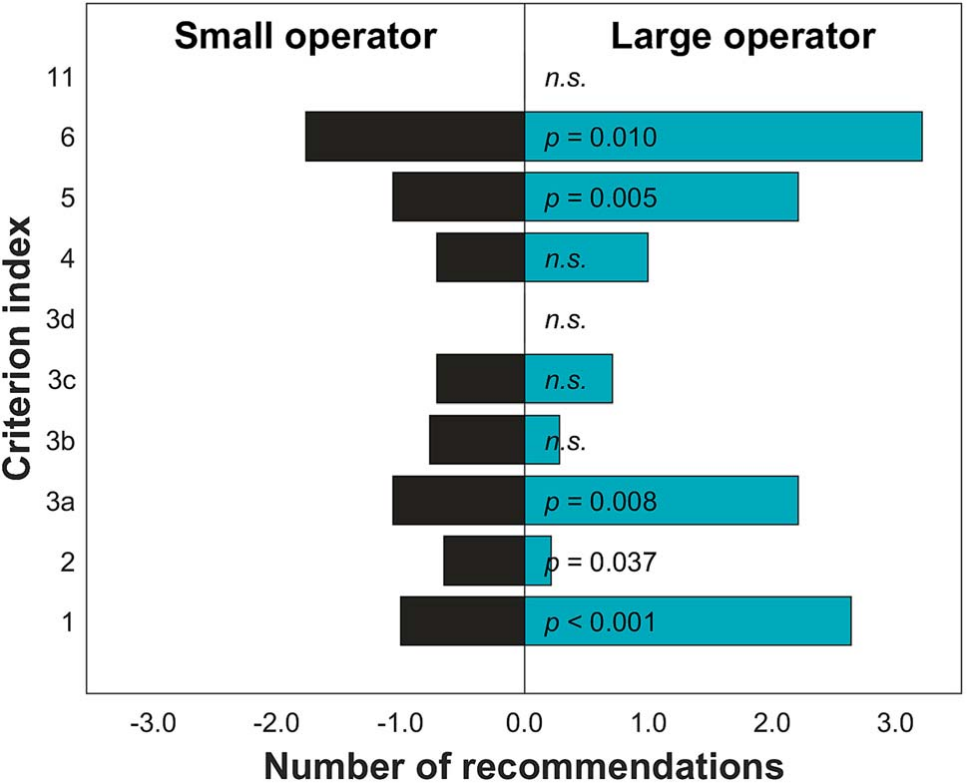
Comparison of the number of recommendations between the large and the small operators within each evaluated criterion. The bars indicate the average number of recommendations received per operator within the sector. Significant differences have been indicated with the *P*-values shown; while the non-significant differences have been indicated with *n.s.* the criterion index in the vertical axis refers to those shown in Table 1.

When looking into the results of categorized criteria (Table 1), it can be noted that in large or public organizations, development needs are particularly focused on the *realization of defined authorizations and responsibilities in practice* (1), *instructions and practices for the execution of examinations, treatment, and procedures* (3a), *personnel training* (5), and on *quality assurance practices and continuous improvement* (6). The public sector received significantly more recommendations in criteria 1, 3a, 5, and 6 than the private sector, while the private sector did not receive more recommendations than the public sector in any criterion (Fig. 2). The greatest difference was observed in criterion 6 related to quality assurance. When comparing the large and small operators, large ones received significantly more recommendations in criteria 1, 3a, 5, and 6 than the small operators again, the greatest difference being observed in criterion 6. Instead, small operators received more recommendations to criterion 2 related to *the practice and flow of information followed in the justification assessment* than the large operators (Fig. 3).

**Figure 4:**
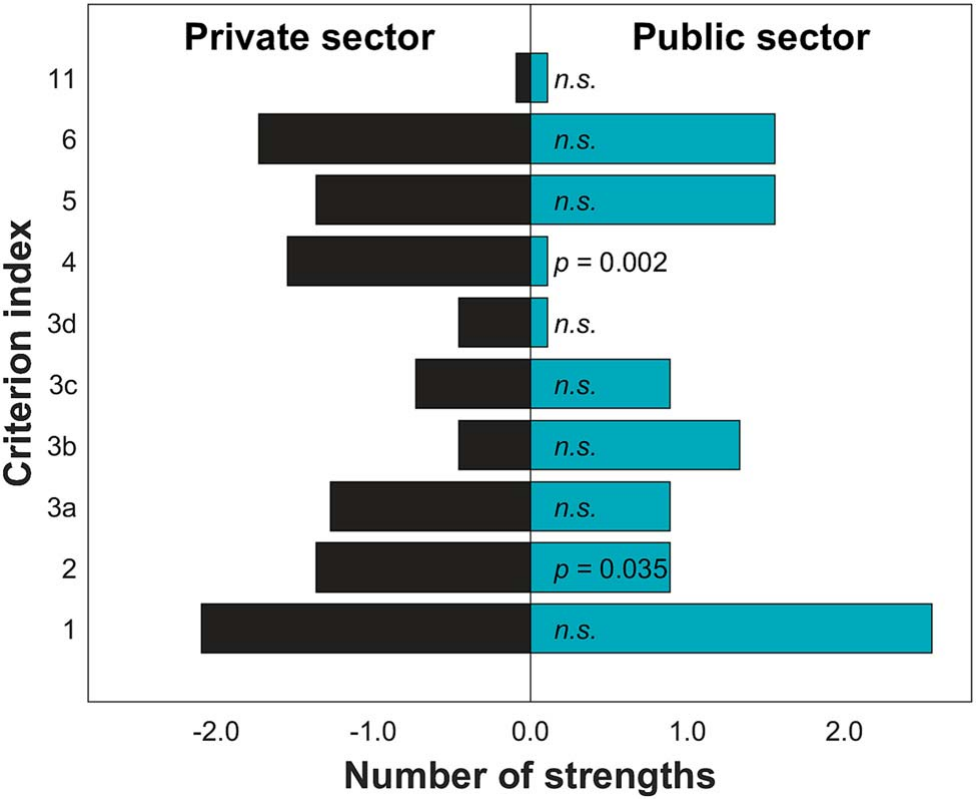
Comparison of the number of strengths between the public and the private sector operators within each evaluated criterion. The bars indicate the average number of strengths identified per operator within the sector. Significant differences have been indicated with the *P*-values shown; while the non-significant differences have been indicated with *n.s.* the criterion index in the vertical axis refers to those shown in Table 1.

Similar comparison on the strengths revealed that private sector operators were found to have significantly more strengths in the criteria regarding the practice and information flow followed in the eligibility assessment (2) , as well as the research and treatment results achieved and information flow (4) (Fig. 4). Public sector operators did not prove to have any strength better than the private sector.

**Figure 5:**
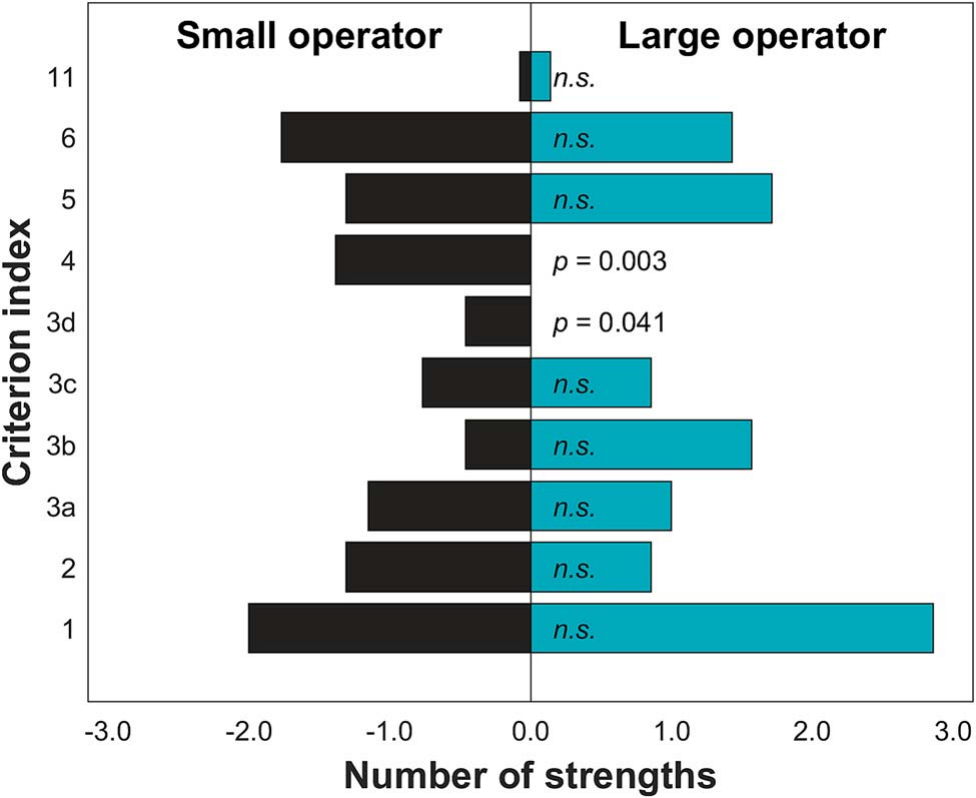
Comparison of the number of strengths between the large and the small operators within each evaluated criterion. The bars indicate the average number of strengths identified per operator within the sector. Significant differences have been indicated with the *P*-values shown; while the non-significant differences have been indicated with *n.s.* the criterion index in the vertical axis refers to those shown in Table 1.

Small operators demonstrated significantly more strengths in the criteria related to the quality of the research statement (3d) and the achieved research and treatment results and information flow (4) (Fig. 5). In no criterion were strengths identified more in large operators than in small operators.

**Figure 6:**
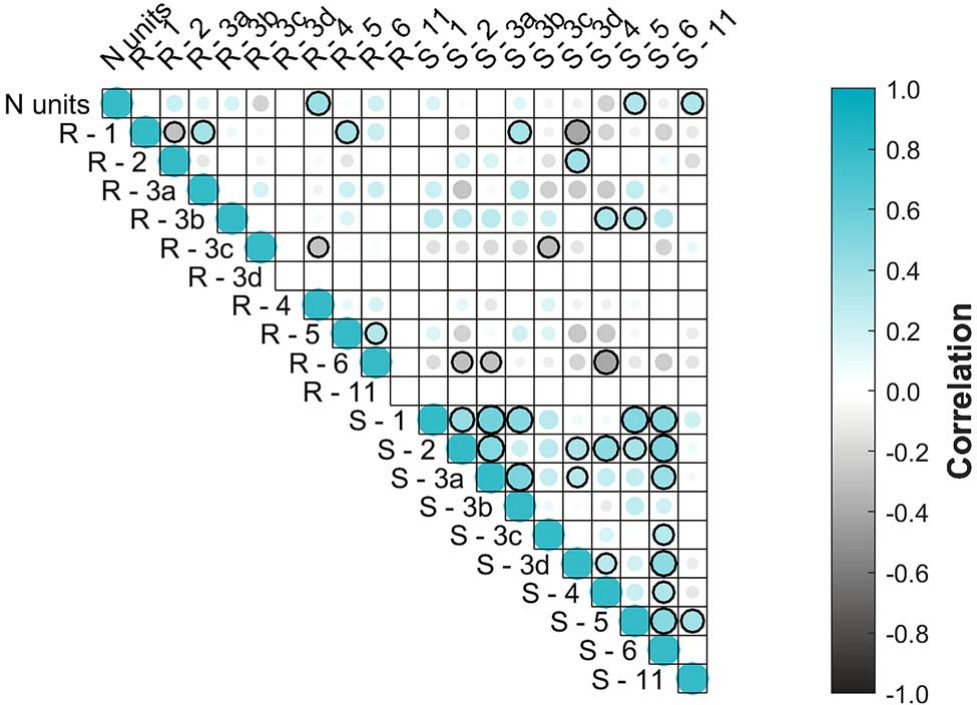
Correlation matrix between the number of audited units (N units), number of recommendations (R) given on each criterion and number of strengths (S) identified on each criterion. The criterion indexes correspond to those described in Table 1. The color and circle size reflect the correlation in a linear manner. The significant (*P* < .05) correlations, excluding the self-correlations, are indicated with the black edge on the circles. No adjustment of *P*-values was made for multiple comparisons.

The overall number of recommendations received did not correlate with the number of audited units within an operator; however, within the criteria of recommendations, criterion 4 displayed a significant correlation with the number of audited units (*r* = 0.485, *P* = .006). In a similar manner the overall number of strengths received did not correlate with the number of audited units within an operator; however, within the criteria of strengths, the criterions 5 and 11 displayed a significant correlation with the number of audited units (*r* = 0.623, *P* = .003 and *r* = 0.518, *P* = .019, respectively). The number of identified strengths correlated with the recommendations received (*r* = 0.258, *P* < .001). Within the criteria, the criterions 2 and 3c displayed a significant correlation with the number of audited units (*r* = 0.471, *P* = .036 and *r* = −0.514, *P* = .021, respectively). The full correlation matrix is displayed in Fig. 6.

## Discussion

In the present study, we reported and analyzed external clinical audit reports from majority of audits conducted on radiological operators in Finland after the implementation of BSSD into national legislation. The data was collected from the fourth clinical audit cycle since the inclusion of the external clinical audits in the Finnish legislation. The results provided here characterize the identified strengths and recommendations given during the audits, and present comparison between different operators. We report that, upon grouping the data into small and large radiological operators, overall more recommendations were received by large radiological operators. When grouping the data into public and private sector operators, the public sector operators also received more recommendations. In addition, distinct differences were found when comparing the number of strengths and recommendations in different evaluation categories (Table 1). While this is more descriptive of the outcome of the clinical audits when comparing the different size operators, the types of feedback provided from the audits tended to differ not just in amount but in context (Fig. 3).

The differences between public and private operators can be explained partly by the more demanding tasks assigned to the public sector operators in Finland with centralized specialized care to University Hospitals, and no selective patient inflow, or when outsourcing to the private sector, less difficult cases are referred to the private sector services, while mostly routine examinations are outsourced [11]. Thus, public sector may outsource radiological examination to the private sector operators providing regional services to the public sector for supporting its demands to provide sufficient level of service [12]. In the private sector, patients often choose to use their radiological services, and are referred there by themselves, by private physicians, by their insurance companies or occupational health practitioners. This causes a great difference in the operational requirements between the public and private sector operators that provide radiology diagnostic services. These characteristic differences may be observed in the radiological procedures conducted and procedure and examination outcomes reflected in the observed differences. Here, we observed that overall, more recommendations were given to the public sector operations for improving practices, and these were more specially emphasized in quality assurance, personnel training, used instructions and practices, and the definition of authorities and responsibilities (Fig. 2). Instead, more strengths were reported with the private sector in association with examination and treatment outcomes as well as flow of information (Fig. 4). These differences may also reflect organizational structure differences with the public operators being mostly considered large operators and the private organizations as small operators. The private organizations may have multiple small units scattered around Finland, while the public organizations tend to be more centralized. The organizational structure could explain the differences in flow of information; however, this is contradicted by the finding that also small operators received more recommendations regarding the information flow than the large operators (Fig. 3).

Private radiology services participate in clinical audits in Europe as a mandatory practice in 17 countries and on voluntary bases in 5 countries, and in 5 countries they do not participate [9], Finland being among those with mandatory participation. One aspect to note that in Finland, no consent is needed from the patients to access data during external clinical audits.

In the present, the first cycle of external clinical audits since the implementation of BSSD was analyzed and no follow-up data was available to quantitatively evaluate the impact of the given recommendations. That data will be made available within the following eight years warranting for impact studies in the coming years. The number of conducted external audits at the time was limited by the number of radiological operators in Finland participating this cycle of the external clinical audits. However, this is currently the largest set of external clinical audit results summarized and published from the Finnish system after the implementation of the BSSD in the national legislation. At this point, there is no national nor European-wide external clinical audit archive for centralized follow-ups, which could enable an efficient evaluation of the impact of the external clinical audits on radiological practices and potentially on safety of the radiological examinations and procedures.

The European QuADRANT project provides a structure for the external clinical audits, which mostly agrees with the structure of the Finnish auditing system in radiological procedures; however, in Finland, there is no regulated audit for internal audit with external direction [9]. Instead, the Finnish audits rely on the internal audits (with internal direction) and the external audits. The internal audits in Finland are conducted in periods of four years and external clinical audits every six to eight years apart from low-level radiation risk practices, such as intraoral dental radiological practices, which are exempted from external clinical audits.

At present, there is no mandated follow-up audit, but it can be recommended by the auditing team. The audits themselves consist of entrance briefing, review, and exit briefing [4]. This procedure results in the reported strengths and recommendations that were counted for this study from each audited radiological operator. As this is structured procedure, follow-ups could be conducted and such studies warranted, not only in Finland but within the EU.

## Conclusions

The present study reported the first experiences from the external audits conducted on clinical radiological practices in Finland after the implementation of BSSD in national legislation. We reported a wide structured analysis on the types of observations made during the external clinical audits and demonstrated some of the characteristic differences observed when auditing different size and type of operators; the large organizations are often public sector operators. In general, our findings indicated that while justification assessments and the quality of clinical studies were generally found to be strong, special attention should be paid to the quality management system procedures, specifically study processes and work instructions, practices for continuous improvement, and staff training. Addressing these areas effectively would require more extensive longitudinal monitoring in clinical audits. By this report, we hope to motivate follow-ups on external clinical audits to enable analyses of the impact that these audits have on the radiological examinations and procedures between the audit cycles.
